# Ag Nanoparticles/α-Ag_2_WO_4_ Composite Formed by Electron Beam and Femtosecond Irradiation as Potent Antifungal and Antitumor Agents

**DOI:** 10.1038/s41598-019-46159-y

**Published:** 2019-07-09

**Authors:** M. Assis, T. Robeldo, C. C. Foggi, A. M. Kubo, G. Mínguez-Vega, E. Condoncillo, H. Beltran-Mir, R. Torres-Mendieta, J. Andrés, M. Oliva, C. E. Vergani, P. A. Barbugli, E. R. Camargo, R. C. Borra, E. Longo

**Affiliations:** 1 CDMF, LIEC, Chemistry Department of the Federal University of São Carlos - (UFSCar), P.O. Box 676, 13565-905 São Carlos, SP Brazil; 2 LIA, Laboratory of Applied Immunology, Department of Genetics and Evolution, Federal University of São Carlos - (UFSCar), São Carlos, Brazil; 3 GROC∙UJI, Institut de Noves Tecnologies de la Imatge (INIT, University Jaume I (UJI), Castelló, 12071 Spain; 4 Department of Inorganic and Organic Chemistry, University Jaume I (UJI), Castelló, 12071 Spain; 5 Institute for Nanomaterials, Advanced Technologies and Innovation, Technical University of Liberec, Studentská 1402/2, 461 17 Liberec, Czech Republic; 6 Department of Analytical and Physical Chemistry, University Jaume I (UJI), Castelló, 12071 Spain; 7 São Paulo State University (UNESP), Department of Dental Materials and Prosthodontics, School of Dentistry at Araraquara, Rua Humaitá, 1680, 14801-903 Araraquara, SP Brazil

**Keywords:** Biomaterials, Cancer

## Abstract

The ability to manipulate the structure and function of promising systems via external stimuli is emerging with the development of reconfigurable and programmable multifunctional materials. Increasing antifungal and antitumor activity requires novel, effective treatments to be diligently sought. In this work, the synthesis, characterization, and *in vitro* biological screening of pure α-Ag_2_WO_4_, irradiated with electrons and with non-focused and focused femtosecond laser beams are reported. We demonstrate, for the first time, that Ag nanoparticles/α-Ag_2_WO_4_ composite displays potent antifungal and antitumor activity. This composite had an extreme low inhibition concentration against *Candida albicans*, cause the modulation of α-Ag_2_WO_4_ perform the fungicidal activity more efficient. For tumor activity, it was found that the composite showed a high selectivity against the cancer cells (MB49), thus depleting the populations of cancer cells by necrosis and apoptosis, without the healthy cells (BALB/3T3) being affected.

## Introduction

Silver nanoparticles (Ag NPs), are considered one of the most important members of the noble metal NPs family. The ever-increasing research activity around them relies on their unique physical, chemical, and biological properties toward applications in catalysis for reduction, oxidation, and oxidative coupling reactions^1–3^, air and water purification systems^4–6^, development of consumer products (e.g., cosmetics, paints, laundry detergents, toys, accessories, and a variety of household applications)^7,8^, and fabrication of sensing devices^9,10^. Moreover, they exhibit diverse useful bioactivities for healthcare^11–13^, antiviral properties^14,15^, bactericidal behavior^16,17^, antifungal activity^18^, as high antimicrobial agent against yeasts, molds, Gram-positive and Gram-negative bacteria^19–23^, anti-cancer^24–26^, and anti-inflammatory effects^27^. Recently, Chernousova and Epple^17^ reviewed the state of research on the effects of Ag on bacteria, cells, and higher organisms, which has shown promising results. However, apart from its outstanding single-NP behavior, its hybridization with semiconductors is also leading to unprecedented behaviors and features that have now paved the way toward promising applications in many fields^28–30^.

The development and use of hybrid nanomaterials, composed by a noble metal–semiconductor, i.e., NPs of Ag, Au, or Pt deposited on a semiconductor surface, have led to advantageous features related to surface plasmon resonance effects, a Schottky contact additional active sites and/or presence of electronics traps^31–33^, that in the end are used to enhance localized heating in living systems, wich may find potential applications in thermal ablation therapies or drug delivery mechanisms^34^.

In this context, a highly attractive semiconductor for its photochemical activity is α-Ag_2_WO_4_. This is an n-type semiconductor with a band gap value of 3.1 eV (400 nm)^34–36^ which has been widely exploited in applications such as visible-light photocatalysis for the degradation of organic dyes and aromatic organic compounds^37–40^, as well as ozone and acetone gas sensing^41,42^. Ag NPs on the semiconductor’s surface could enhance the surface plasmon resonance effect, which might improve the separation rate of the photo-generated holes and electrons in the composite, leading to augmented photo-derived phenomena. Therefore, developing a metal–semiconductor junction of Ag/Ag_2_WO_4_ in a controllable way would represent a wise strategy that may have significant potential for the design of novel materials^43^.

The great potential for growing Ag NPs on the framework of semiconductors, induced by electron beam irradiation, is well known^44–46^. Recently, our research group demonstrated that the exposure to electron or femtosecond (fs) laser beams can give rise to many fascinating and unexpected phenomena, such as the formation and growth process of Ag NPs on α-Ag_2_WO_4_ crystals^34,47–52^ with a wide range of applications, including microbial^53,54^ and antifungal^55^ agents. Very recently, we demonstrated that the interaction between pulsed fs laser irradiation and α-Ag_2_WO_4_ revealed a new processing alternative for the formation of Ag NPs on α-Ag_2_WO_4_ with bactericidal properties^36^. From a medical point of view, the development of novel composites as antimicrobials is very attractive, owing to the worldwide crisis of bacterial resistance to conventional, narrow-target antibiotics.

Taking into consideration its afore mentioned biological relevance, the synthesis of Ag NPs/α-Ag_2_WO_4_ composite by electron beam and fs irradiation was undertaken and a systematic study of its antifungal and antitumor activities was carried out in order to analyze the beneficial implications of the composite in two of the most important bio-medical applications (as an antitumor and antifungal agent) that directly impact the healthcare of human kind.

## Results

### Structural analysis

Four samples were studied: pure α-Ag_2_WO_4_, a composite irradiated with electrons (Ag NPs/α-Ag_2_WO_4_:E), a composite irradiated by a fs laser beam under non-focused conditions (Ag NPs/α-Ag_2_WO_4_:NF), and a composite irradiated by a fs laser beam under focused conditions (Ag NPs/α-Ag_2_WO_4_:F).

**Figure 1:**
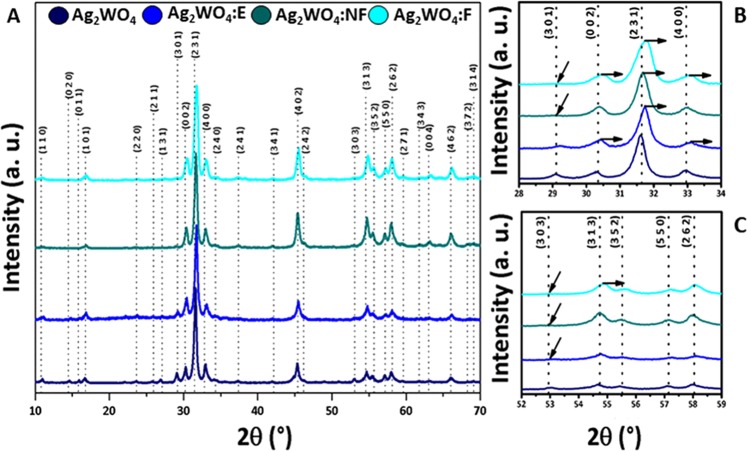
(**A**) X-ray diffractograms of the α-Ag_2_WO_4_ samples before and after the different irradiations; (**B**) Approximation of the diffracted region between 28 and 34° and (**C**) Approximation of the diffracted region between 52 and 59°.

Figure 1A shows the X-ray diffraction (XRD) patterns of the samples to evaluate the crystalline order/disorder at long distances. The samples present an orthorhombic structure with a space group of *Pn2n* and eight molecules per unit cell (Z = 8), according to the crystallographic data sheet n° 2489692 in the Inorganic Crystal Structure Database (ICSD). The lattice parameters are: a = 10.878 Å, b = 12.009 Å, and c = 5.89 Å. Figure 1B shows the most intense diffraction peak (231), which was shifted slightly to higher values of 2θ for all composites. This behavior is also observed for the (002) and (400) peaks of Ag NPs/Ag_2_WO_4_:E composite, and the (002), (400) and (313) peaks of Ag NPs/Ag_2_WO_4_:F composite (Fig. 1C). Another phenomenon that occurs is the suppression of certain peaks in relation to the non-irradiated phase. For all composite, the (303) peak is not observed, whereas for the Ag NPs/Ag_2_WO_4_:F composite, the peak (301) does not appear (Fig. 1B).

**Figure 2:**
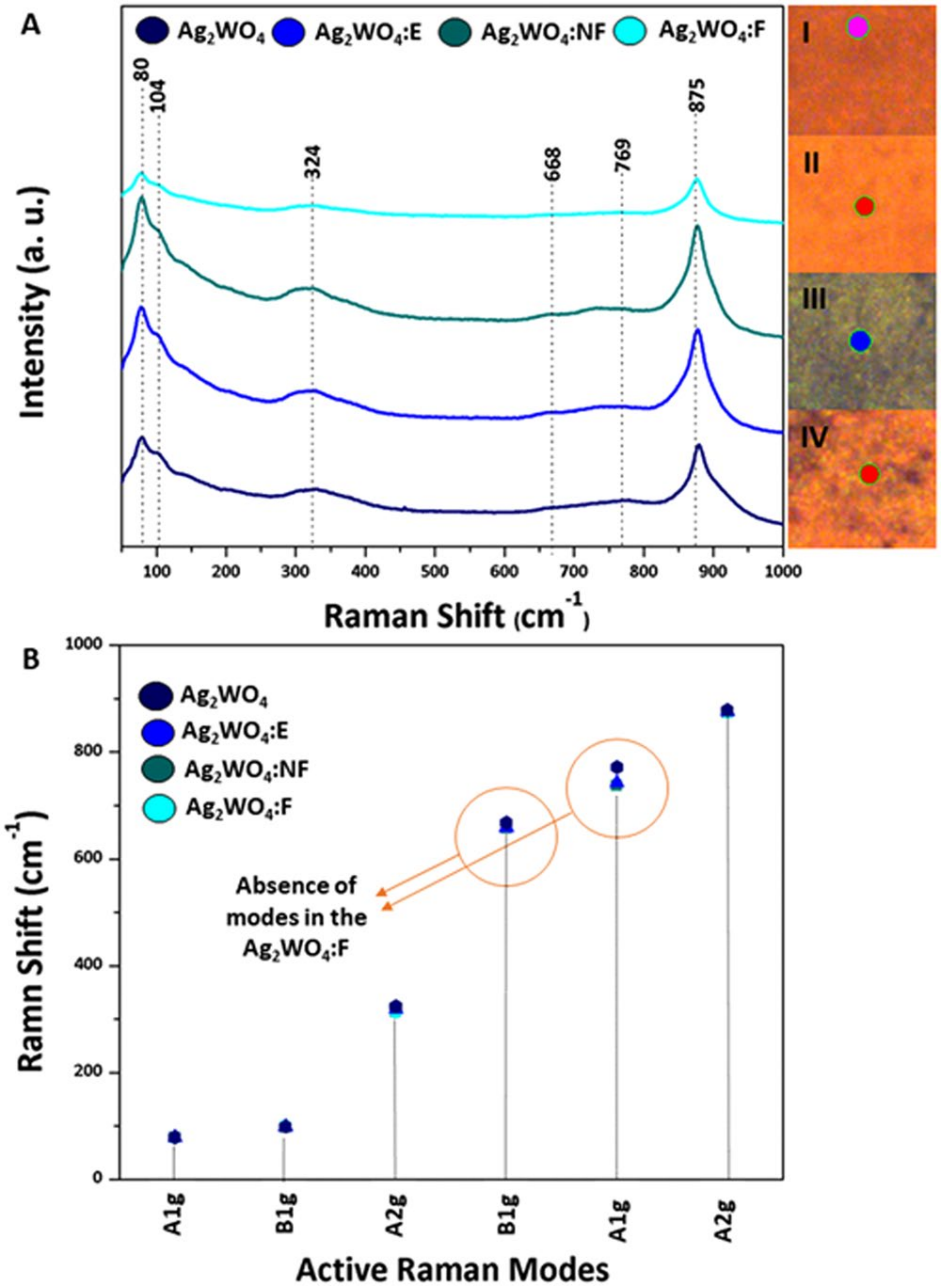
Micro-Raman spectra of α-Ag_2_WO_4_ samples before and after the different irradiations with the respective regions in the sample (I–iv); (**B**) Comparison between relative positions of experimental Raman-active modes for the samples.

Micro-Raman spectroscopy analysis, as shown in Fig. 2, was performed to determine the short-range structural order/disorder effects, and is thus a structural complementary technique to XRD. α-Ag_2_WO_4_ belongs to the C_2v_ symmetry group; its structure is composed by four different clusters associated with the local coordination of Ag: [AgO_2_], [AgO_4_], [AgO_6_], and [AgO_7_], and only one cluster of W: [WO_6_]. The active Raman modes are observed at 80, 104, 324, 668, 769, and 875 cm^−1^ (Fig. 2A). The modes located at lower wavelengths, 80 and 104 cm^−1^, can be associated to a transition A_1g_ of the crystal lattice modes of Ag^53,56^ that do not undergo changes, even for samples irradiated by laser and electrons. The other modes correspond to movements of the [WO_6_] cluster, the values 875 cm^−1^ and 769 cm^−1^ are associated with the A_2g_ and A_1g_ vibrational modes of the symmetric and asymmetric stretching modes of O-W-O, respectively^53,56,57^. The B_1g_ mode of 668 cm^−1^ was related to stretching modes of the W-O bond and the A_2g_ mode at 324 cm^−1^ was attributed to cationic lattice vibrations^57^. It is observed that both Ag NPs/α-Ag_2_WO_4_:E and Ag NPs/α-Ag_2_WO_4_:NF composites do not present significant changes, whereas for the Ag NPs/α-Ag_2_WO_4_:F composite, the defined peaks at 668 and 769 cm^−1^ disappear, thus presenting a higher structural disorder at short-range than the other composites (Fig. 2B).

**Figure 3:**
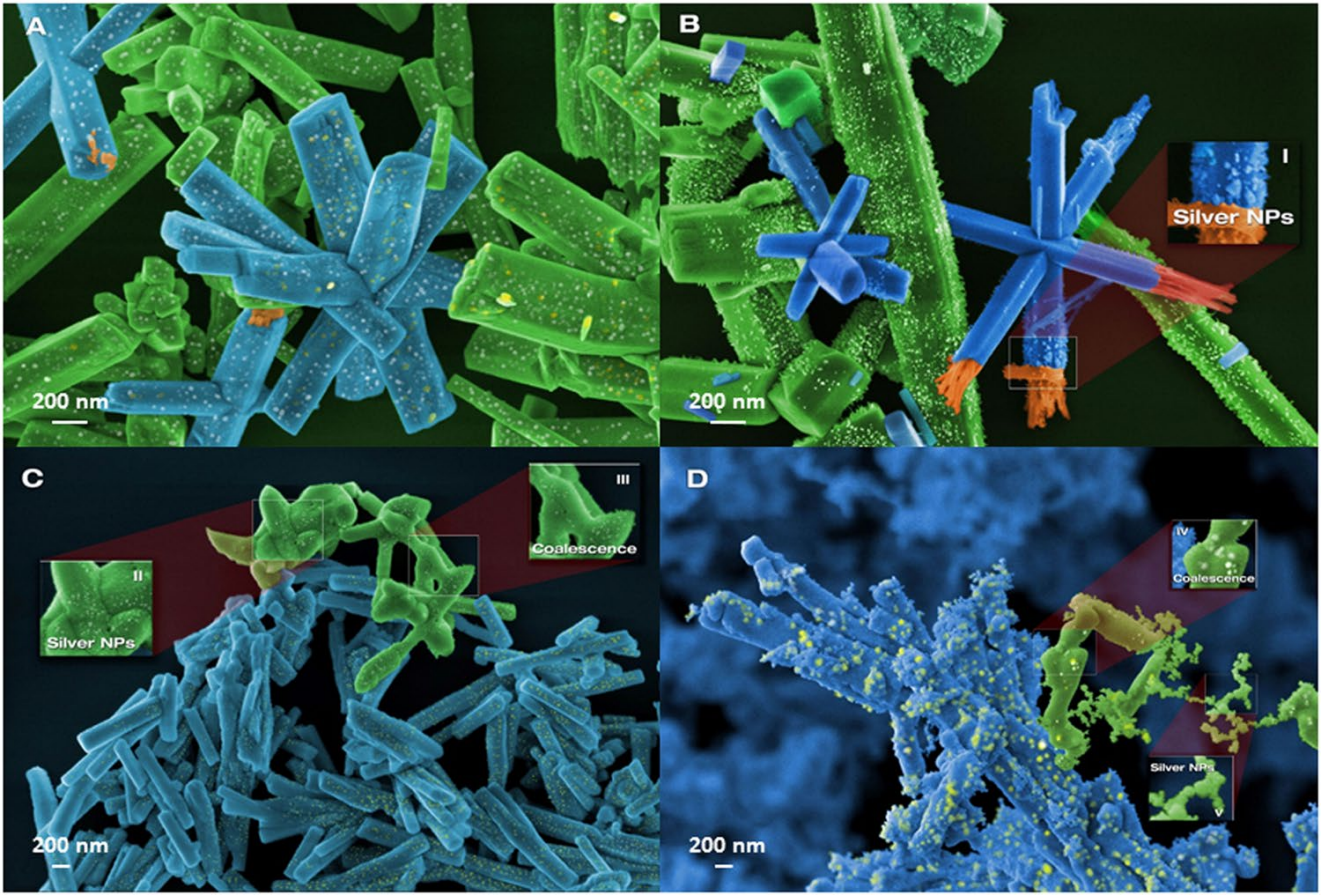
FE-SEM images for (**A**) α-Ag_2_WO_4_; (**B**) α-Ag_2_WO_4_:E (**C**) α-Ag_2_WO_4_:NF; (**D**) α-Ag_2_WO_4_:F.

Figure 3 presents images of the samples obtained by field emission scanning electron microscopy (FE-SEM). In the α-Ag_2_WO_4_ sample (Fig. 3A), the formation of Ag NPs was not observed because the system was not disturbed by any external energy source. For Ag NPs/α-Ag_2_WO_4_:E composite (Fig. 3B), it is possible to observe the delocalized formation of Ag NPs, as reported by Longo *et al*.^34^. Figure 3C,D display the images of Ag NPs/α-Ag_2_WO_4_:NF and Ag NPs/α-Ag_2_WO_4_:F composite, respectively. In the Ag NPs/α-Ag_2_WO_4_:NF composite, there is a small number of Ag NPs attached to the semiconductor’s surface (similar to Ag NPs/α-Ag_2_WO_4_:E composite) and a slight sinterization of α-Ag_2_WO_4_ microrods, whereas the Ag NPs/α-Ag_2_WO_4_:F composite presents many spherical Ag NPs with a larger particle size than in α-Ag_2_WO_4_:NF, probably due to an enhancement of the sinterization process experienced by α-Ag_2_WO_4_ microrods, as was observed by Andrés *et al*.^34^.

All materials have a normal size distribution, and the obtained sizes of width and length for the samples are of 0.31 ± 0.02 μm and 2.76 ± 0.88 μm, 0.33 ± 0.03 μm and 2.83 ± 0.62 μm, 0.33 ± 0.11 μm and 2.32 ± 1.2 μm, 0.36 ± 0.13 μm and 2.54 ± 0.95 μm for Ag_2_WO_4_, Ag_2_WO_4_:E, Ag_2_WO_4_:NF and Ag_2_WO_4_:F respectively. The increase in the error of the samples irradiated with the laser in femtoseconds, is due to the sinterization of some rods of α-Ag_2_WO_4_, as shown in the Fig. 3C,D.

### Antifungal activity

**Figure 4:**
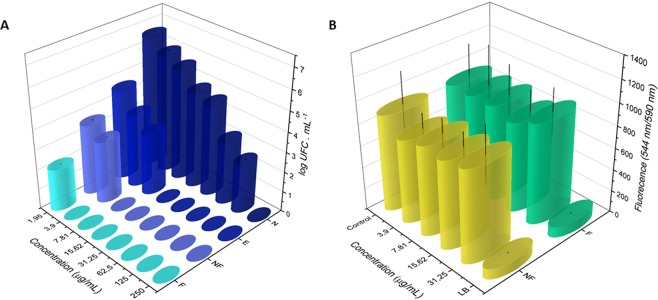
*Candida albicans* (**A**) and FGH (**B**) growth as a function of different concentrations of samples.

The antifungal activity of the samples was evaluated against the biofilm formation of the *Candida albicans* ATCC 90028 reference strain. The minimum obtained inhibitory concentration (MIC) and minimum fungicidal concentration (MFC) values are presented in the Fig. 4A. The results obtained by counting colony forming units per mL (CFU.mL^−1^) show that the most effective samples were, in descending order: Ag NPs/α-Ag_2_WO_4_:F, Ag NPs/α-Ag_2_WO_4_:NF, Ag NPs/α-Ag_2_WO_4_:E, and α-Ag_2_WO_4_. The highest concentration used for the tests was 125.00 μg/mL and we observe that all irradiated materials have antifungal activity. Even at sub-inhibitory concentrations, the reduction in the number of viable fungal colonies is observed in relation to the control. A dose-dependent effect was observed.

**Figure 5:**
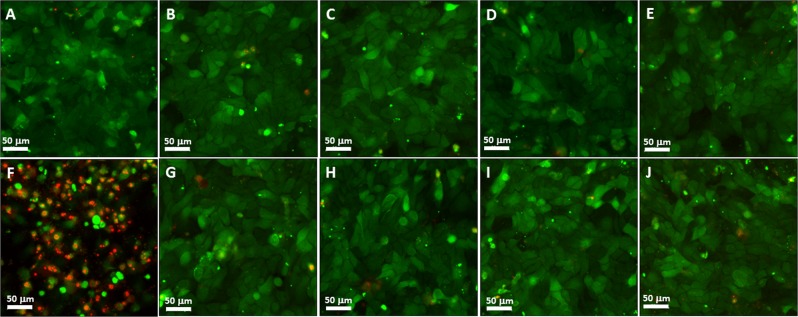
Fluorescence staining of FGH cells after α-Ag_2_WO_4_ microcrystal incubation. (**A**) Control (without microcrystal); (**B**) 31.25 μg/mL α-Ag_2_WO_4_:NF; (**C**) 15.62 μg/mL α-Ag_2_WO_4_:NF; (**D**) 7.81 μg/mL α-Ag_2_WO_4_:NF; (**E**) 3.90 μg/mL α-Ag_2_WO_4_:NF; (**F**) Death control (CAM); (**G**) 31.25 μg/mL α-Ag_2_WO_4_:F; (**H**) 15.62 μg/mL α-Ag_2_WO_4_:F; (**I**) 7.81 μg/mL α-Ag_2_WO_4_:F; (**J**) 3.90 μg/mL α-Ag_2_WO_4_:F.

**Figure 6:**
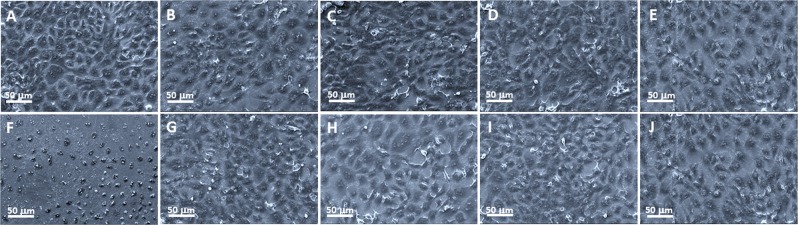
Scanning electron microscopy as a confirmation of biocompatibility α-Ag_2_WO_4_ effects on FGH cell morphology. (**A**) Control (without microcrystal); (**B**) 31.25 μg/mL α-Ag_2_WO_4_:NF; (**C**) 15.62 μg/mL α-Ag_2_WO_4_:NF; (**D**) 7.81 μg/mL α-Ag_2_WO_4_:NF; (**E**) 3.90 μg/mL α-Ag_2_WO_4_:NF; (**F**) Death control (LB); (**G**) 31.25 μg/mL α-Ag_2_WO_4_:F; (**H**) 15.62 μg/mL α-Ag_2_WO_4_:F; (**I**) 7.81 μg/mL α-Ag_2_WO_4_:F; (**J**) 3.90 μg/mL α-Ag_2_WO_4_:F.

Four concentrations of Ag NPs/α-Ag_2_WO_4_:NF and Ag NPs/α-Ag_2_WO_4_:F composites were studied on a human gingival fibroblasts (FGH) cell line, because this exhibiting better fungicidal activity. After 24 h of incubation, the effects of these composites on cell viability, cell proliferation, and cell morphology were evaluated by the resazurin assay (Alamar Blue) quantitative fluorimetric assay (Fig. 4B), confocal laser scanning microscopy (CLSM) (Fig. 5), and SEM (Fig. 6), respectively. The studied concentrations were 31.25, 15.62, 7.81, and 3.90 μg/mL, respectively, because they are above the optimum range of fungicidal activity of the materials. The results show no statistically significant loss of cell viability for this concentrations compared to the control (CT), which shows a contrasting behavior with respect to cell death control treated with Triton-X 100 0.9% buffer (LB) (see Fig. 4B). The proliferation assay on CLSM by carboxyfluorescein succinimidyl ester and propidium iodide (PI) showed a homogeneous label of CFSE by HGF cells for the concentrations, whereas no important staining with PI was detected. This was very similar to the CT cells, and completely different from the pattern of the fluorescence cells treated with the cell death control (CAM), showing no influence of α-Ag_2_WO_4_:NF and α-Ag_2_WO_4_:F materials on HGF cell proliferation (see Figs 5 and 6). The cell morphology observed by SEM showed the presence of whole cells, with integrated shape and attached to the substrate, similar to the CT. Even at 31.25 µg/mL, the cells maintained their normal morphology, showing no signs of typical morphology death as observed for cells treated with LB (see Fig. 6).

**Figure 7:**
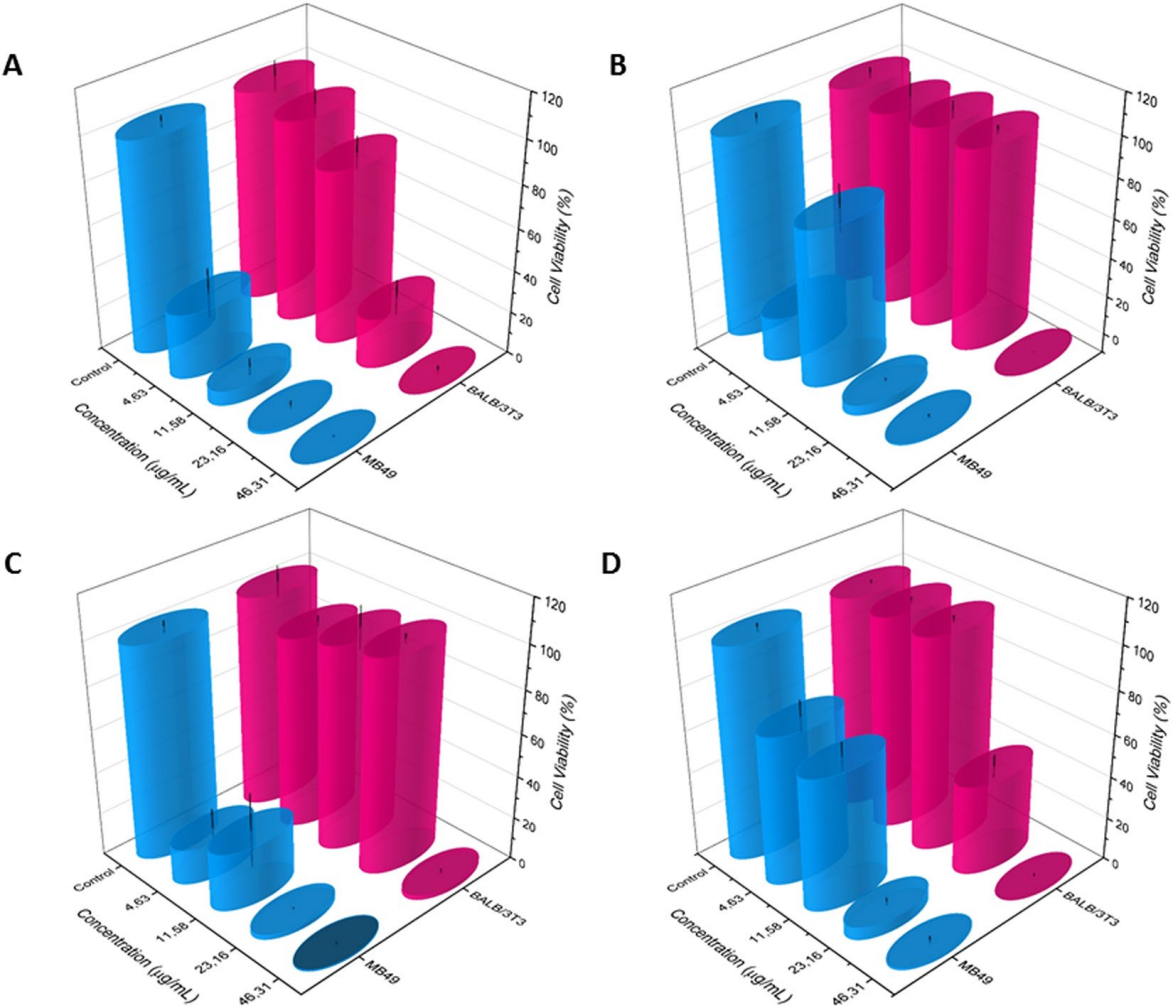
Viability assay of MB49 and BALB/3T3 cells on exposure to samples at concentrations 4.63; 11.58; 23.16; 46.31 µg/ml respectively, for 24 h. (**A**) Ag_2_WO_4_; (**B**) Ag_2_WO_4_:E; (**C**) Ag_2_WO_4_:NF; (**D**) Ag_2_WO_4_:F.

In this study, we analyzed the viability of the MB49 and BALB/3T3 cells exposed for 24 h to different samples at concentrations of 1.0 (4.63 µg/mL), 2.5 (11.58 µg/mL), 5.0 (23.16 µg/mL), and 10.0 µmol/mL (46.31 µg/mL). MB49 is an induced bladder carcinoma derived from mouse, and BALB/3T3 is a representative of no tumorigenic cells evaluated by the resazurin assay (Alamar Blue) quantitative fluorimetric assay (Fig. 7). In relation to the major effect of the α-Ag_2_WO_4_, the BALB/3T3 cell culture presented 95% and 80% cell viability when exposed to concentrations of 4.63 and 11.58 µg/mL, respectively, whereas the MB49 cancer cells presented 50% and 10% of cell viability in comparison to the CT (Fig. 7A). In relation to the other concentration, the loss of viability was intense for both types of cells, mainly at a dose of 46.31 µg/m. For Ag NPs/α-Ag_2_WO_4_:E composite, the BALB/3T3 cell culture at 4.63, 11.58, and 23.16 µg/mL concentrations presented viability equivalent to the CT group, whereas the MB49 cell presented a significant reduction in the viability (20%, 80%, and 10%) (Fig. 7B). This increase in cell viability at the intermediate concentration of 11.58 μg/mL shows that this sample has a non-linear behavior against MB49 tumor cell exposure, thus presenting a minimum of inhibition different from the minimum concentration, obeying a gaussian behavior.

For Ag NPs/α-Ag_2_WO_4_:NF composite, the BALB/3T3 cell culture exposed to 4.63, 11.58, and 23.16 µg/mL concentrations of the composite presented viability equivalent to the CT in contrast with the MB49 culture that showed a significant reduction (16%, 32%, and 2%) at the same concentrations (Fig. 7C), with perfil of cancer activity very similar with Ag NPs/α-Ag_2_WO_4_:NF composite (gaussian behavior). For Ag NPs/α-Ag_2_WO_4_:F composite, the BALB/3T3 cells exposed to 4.63 and 11.58 µg/mL of the sample presented viability greater than 95%, whereas the MB49 cell showed viabilities of 64% and 60%, respectively (Fig. 7D).

**Figure 8:**
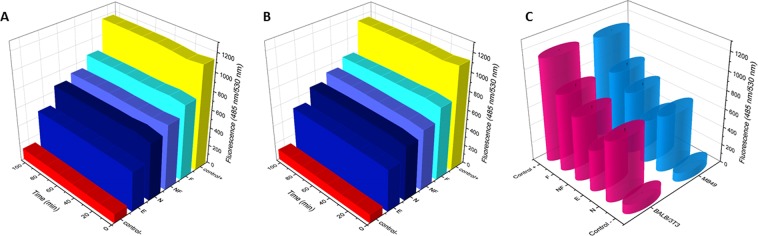
Intracellular detection production of reactive oxygen species (ROS) by BALB/3T3 (**A**) and MB49 (**B**) cells by exposing the samples of Ag_2_WO_4_:F; Ag_2_WO_4_:NF; Ag_2_WO_4_; Ag_2_WO_4_:E, respectively up to 120 min. Comparison of mean of ROS production by cells exposed to samples (**C**).

Figure 8 illustrates the kinetics of the production of the reactive oxygen species (ROS) in the period of 120 min by MB49 and BALB/3T3 exposed to the different samples at a concentration of 23.16 µg/mL (5 µmol/mL) in comparison with positive (1.0 mM of H_2_O_2_) and negative control. For the BALB/3T3 cells, the highest ROS production was reached with the exposure to Ag NPs/α-Ag_2_WO_4_:F followed by Ag NPs/α-Ag_2_WO_4_:NF, α-Ag_2_WO_4_, and Ag NPs/α-Ag_2_WO_4_:E (Fig. 7A). For the MB49 cell line, in the same way, the highest production of ROS was similar to the obtained with BALB/3T3 cells (α-Ag_2_WO_4_:F, α-Ag_2_WO_4_:NF, α-Ag_2_WO_4_, and α-Ag_2_WO_4_:E) (Fig. 8B). In comparison with CT, all materials presented a continuously higher production of ROS during the two hours of measurement. The ROS production pattern over time was equivalent for both cell lines (Fig. 8A,B). The level of ROS of the BALB/3T3 cells did not have a linear relationship with the different level of cytotoxicity of the materials (Fig. 7). To the MB49, the toxicity was high, independent of the level of ROS measured.

**Figure 9:**
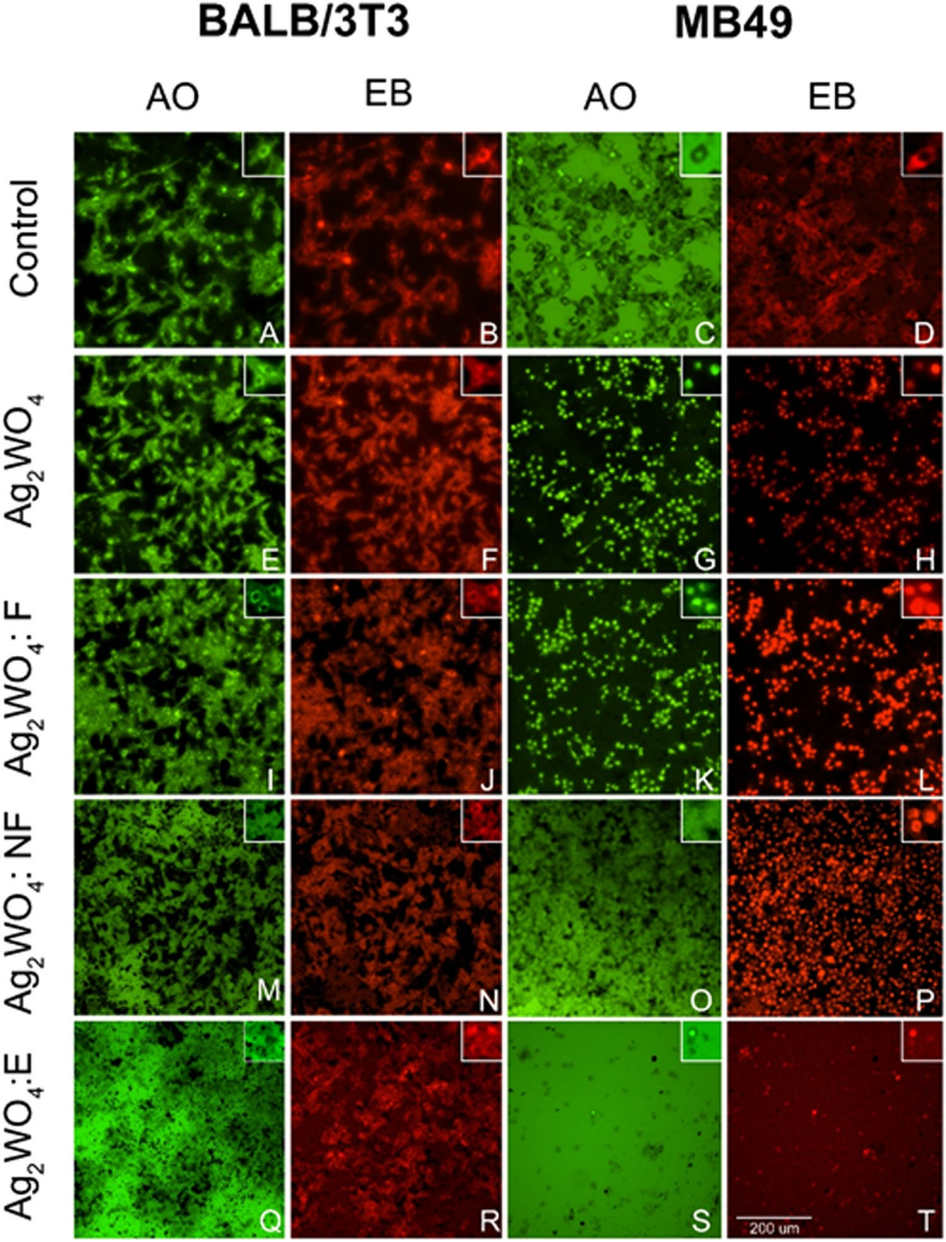
Cell death assay (apoptosis and necrosis). AO/EB: Acridine Orange/Ethidium Bromide. (**A**) control cells BALB/3T3 absent from apoptosis; (**B**) control cells BALB/3T3 absent from necrosis; (**C**) control cells MB49 absent from apoptosis; (**D**) control cells MB49 absent from necrosis; (**E**) Ag_2_WO_4_ cells BALB/3T3 absence amount of apoptosis death; (**F**) Ag_2_WO_4_ cells BALB/3T3 absence of necrotic death; (**G**) Ag_2_WO_4_ cells MB49 large amount of apoptosis death; (**H**) Ag_2_WO_4_ cells MB49 absence of necrotic death; (**I**) Ag_2_WO_4_:F cells BALB/3T3 absence amount of apoptosis death; (**J**) Ag_2_WO_4_:F cells BALB/3T3 absence of necrotic death; (**K**) Ag_2_WO_4_:F cells MB49 large amount of apoptosis death; (**L**) Ag_2_WO_4_:F cells MB49 large amount of necrotic death; (**M**)) Ag_2_WO_4_:NF cells BALB/3T3 absence of apoptosis death; (**N**) Ag_2_WO_4_:NF cells BALB/3T3 absence of necrotic death; (**O**) Ag_2_WO_4_:NF cells MB49 absence of apoptosis death; (**P**) Ag_2_WO_4_:NF cells MB49 large amount of necrotic death; (**Q**) Ag_2_WO_4_:E cells BALB/3T3 absence of apoptosis death; (**R**) Ag_2_WO_4_:E cells BALB/3T3 absence of necrotic death; (**S**) Ag_2_WO_4_:E cell debris (cellular debris) MB49; (**T**) Ag_2_WO_4_:E cell debris (cellular debris).

By analyzing the specific type of death caused by each sample for both cell lines through the acridine orange (AO) and ethidium bromide (EB) assay, it is possible to observe a difference in resistance of the BALB/3T3 and MB49 lines in relation to the type of sample. The fluorescence images (Fig. 9) show that the BALB/3T3 cell (healthy cell) exposed at a concentration of 23.16 µg/mL of α-Ag_2_WO_4_, Ag NPs/α-Ag_2_WO_4_:F, α- Ag NPs/Ag_2_WO_4_:NF, or Ag NPs/α-Ag_2_WO_4_:E maintained its membranes (AO−/EB−). On the contrary, MB49 exposed to α-Ag_2_WO_4_, Ag NPs/α-Ag_2_WO_4_:F, and Ag NPs/α-Ag_2_WO_4_:NF presented signals of apoptosis (Fig. 9G,H: AO+/EB−), late apoptosis (Fig. 9K,L: AO+/EB+) and necrosis (Fig. 9O,P: AO−/EB+), respectively. The cells exposed to Ag NPs/α-Ag_2_WO_4_:E totally lost the integrity of the membranes and it was possible to observe only the presence of cellular debris (Fig. 9O,P).

**Figure 10:**
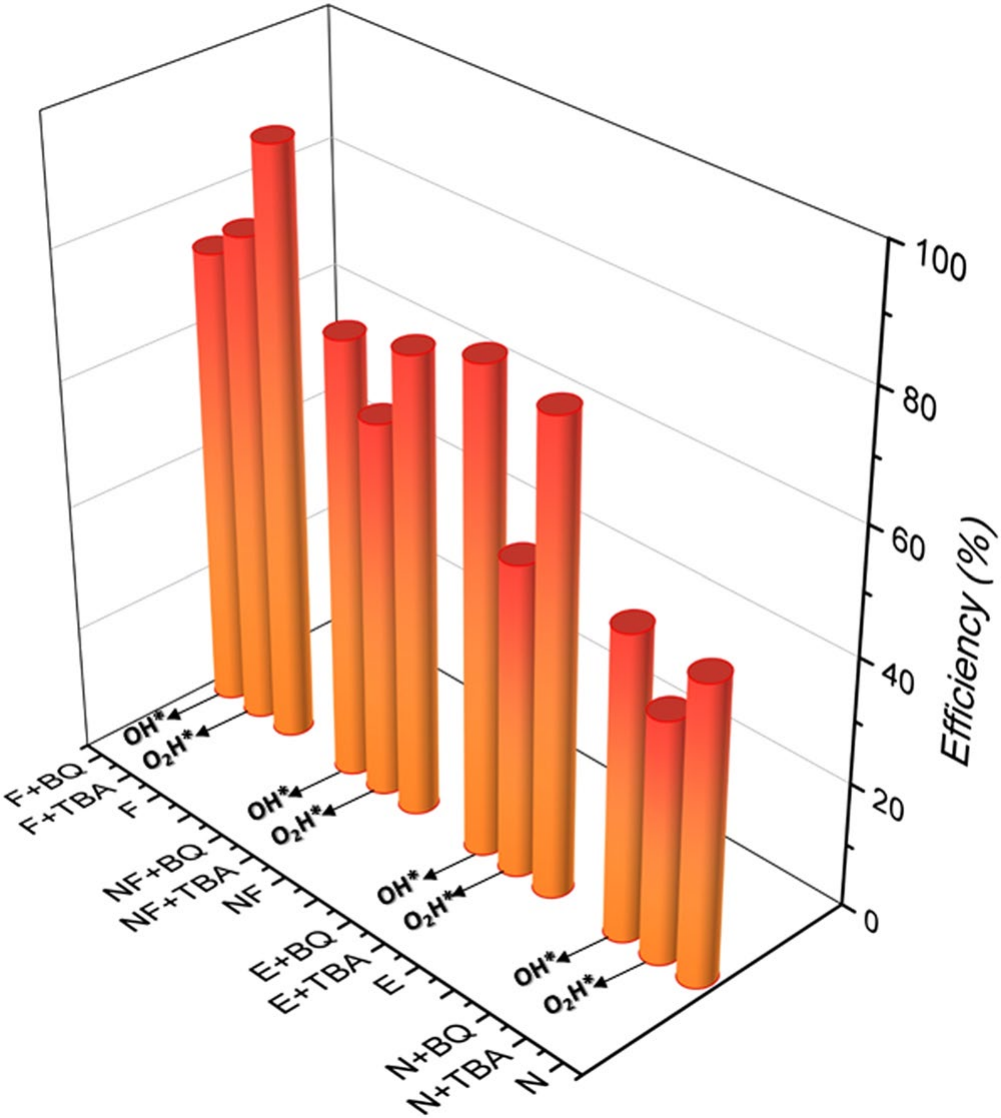
Influence of various scavengers onto the photodegradation of RhB.

In order to evaluate the ROS production of the composites, tests of photocatalytic activity (Fig. 10) were performed with the addition of appropriate reactive species scavengers. For these tests, benzoquinone (BQ) was used to capture superoxide radicals $({O^{\prime} }_{2})$ and tert-butyl alcohol (TBA) was used to capture hydroxyl radicals (OH^*^), because these species are directly linked to the oxidative stress of these cells and their subsequent death. α-Ag_2_WO_4_ presents a low efficiency in the photocatalysis (~48%) and when the TBA is added, this value reduces, indicating an action by the radical OH^*^ in the oxidative processes. The same was done by adding BQ to Ag NPs/α-Ag_2_WO_4_ composite, but the photocatalytic activity remained constant, showing that the ${O^{\prime} }_{2}$ moiety is not generated by α-Ag_2_WO_4_. For Ag NPs/α-Ag_2_WO_4_:NF and Ag NPs/α-Ag_2_WO_4_:E composites, an increase in the dye degradation is observed, owing to the structural modifications of both samples. When TBA is added to the process, a reduction of the photocatalytic efficiency occurs, and once the BQ is added, the efficiency becomes constant, that is, the oxidative process occurs through the OH^*^ radical as well as in the α-Ag_2_WO_4_. Finally, Ag NPs/α-Ag_2_WO_4_:F composite was the only that showed a contribution to the radical ${O^{\prime} }_{2}$, apart from the contribution of OH^*^, thus causing an increase in its photocatalytic efficiency among all the materials.

## Discussion

In the XRD patterns (Fig. 1), we observe a right-shift of some peaks in the composites, including the main peak (231), as well as the suppression of certain diffraction peaks (Fig. 1B,C). This change can be associated with the electron beam irradiation and fs laser irradiation that provokes a decrease in the interplanar distances of this plane with a concomitant small reduction in the volume of the unit cell, indicating that the effect of different irradiations changes certain orientations of the clusters forming the material, increasing the long-range disorder. It is not possible to observe Ag in the XRD spectrum, because the growth phenomena of these Ag NPs are delocalized and superficial in α-Ag_2_WO_4_.

Another factor related to structural order/disorder effects at long-range can be found by analyzing the full width at half maximum of the most intense peak of the XRD patterns, related to the (231) plane. An increment in the FWHM of this peak in the non-irradiated material with respect to the others is observed as 0.34°, 0.40°, 0.43°, and 0.57° related to α-Ag_2_WO_4_, α-Ag_2_WO_4_:E, α-Ag_2_WO_4_:NF, and α-Ag_2_WO_4_:F, respectively. These values indicate that when the material is irradiated with the focused fs laser, the structural disorder is much higher than it is for irradiation with the non-focused fs laser and electron beam.

The micro-Raman spectroscopy analysis shows structural disorder associated with the vibrational modes of [WO_6_] clusters (Fig. 2). The formation of Ag NPs during the irradiation (fs laser and electron beam) provokes distortions in the [WO_6_] clusters. According to Longo *et al*.^34^, when the α-Ag_2_WO_4_ is irradiated with electron beam in a SEM, Ag nanofilamentos grow in the surface of the material, thereby forming a Ag NPs/α-Ag_2_WO_4_ composite (Fig. 3B). This happens because [AgO_2_] and [AgO_4_] clusters accept the incoming electron density and the reduction of Ag cation occurs with a concomitant Ag formation process. When the material is irradiated with a fs laser, spherical Ag NPs grow on the surface of α-Ag_2_WO_4_, yielding a Ag NPs/α-Ag_2_WO_4_ composite (Fig. 3C,D). Fs laser irradiation strips the equilibrium system, causing the segregation of these Ag NPs^36^.

As mentioned above, α-Ag_2_WO_4_ without any irradiation is an n-type semiconductor, it has positive charge holes in its structure, which combined with the effect of the Ag present on the material have a high oxidizing power. Under electron beam irradiation, α-Ag_2_WO_4_ semiconductor becomes n/p due to the formation of Ag vacancies within the material. On the contrary, when α-Ag_2_WO_4_ is subject to fs radiation, the structural disorder of the sample is increased because the fs laser beam is concentrated in localized regions of the material’s surface, promoting an increment in the segregation process of Ag NPs out of α-Ag_2_WO_4_ semiconductor; then, a new semiconductor with a larger p character is formed, owing to a higher density of Ag vacancies.

When α-Ag_2_WO_4_ was irradiated with electron beam and fs laser in non-focused mode, nucleation and formation of Ag NPs was observed, transforming α-Ag_2_WO_4_ into Ag NPs/α-Ag_2_WO_4_ composite, acting as a n/p semiconductor type. In the α-Ag_2_WO_4_: NF sample, not only the plasmonic effect is potentiated but there is selectivity and only the cancer cells are affected under the effects of the new tungsten clusters. This is probably due to the highly unregulated energy rate of tumor cells, since they exhibit aberrant growth rates relative to normal cells, which require high levels of ATP for the production of carbohydrates, proteins, lipids and nucleic acids^58,59^.

The antimicrobial activity of α-Ag_2_WO_4_ composites has been previously described^36,53–55^. Comparing the antimicrobial activity of α-Ag_2_WO_4_ and α- Ag NPs/Ag_2_WO_4_:E composite, a fourfold improvement in the activity against Methicillin-Resistant *Staphylococcus aureus* ATCC 33591 (MRSA) bacteria was observed. In previous works^36^, the antimicrobial action of the Ag NPs/α-Ag_2_WO_4_:F composite against the MRSA bacteria exhibited a 32-fold greater efficacy than the non-irradiated material, where the nucleation of metallic Ag NPs can be associated to a surface plasmonic effect, with a subsequent enhancement of their antimicrobial activity. In the present study, Ag NPs/α-Ag_2_WO_4_:F composite exhibits the best antifungal activity, showing a larger number of defects and metallic Ag NPs on the material surface, which is believed to be the reason for the enhanced surface plasmonic effect leading to a bactericidal improvement (Fig. 4A). The biofilms treated with this sample presented a smaller area of coverage, smaller number of cells, and morphologically altered cells, in comparison with the other biofilms.

The known antimicrobial potential of α-Ag_2_WO_4_ is a notable feature of this material^54^ and its antifungal potential was shown in this work. The development of technologies that provide modifications of different characteristics with respect to their morphological structure as well as highly controlled physicochemical activities and surface area, increases its capacity of application and its therapeutic use^60^. Ag NPs is extremely poisonous against planktonic cells and biofilms of different species, at exceptionally low concentrations, compared to different commercial antimicrobials^61^. Apart from its capacity to participate in oxidative reactions, Ag NPs damages and inhibits Fe–S-containing dehydratases at bactericide concentrations, leading to microbial death^62^.

Previously, Ag NPs have been reported to have effective antitumor effects at low concentrations, as they cause irreversible DNA damage^63^. However, their genotoxic and cytotoxic effects provoked some instability in its use. The combination of Ag NPs with other materials, such as tungsten (W) has led to its reduced toxicity according to different studies^54,55^. As eukaryotic and bacterial cells possess distinct transport metalloproteins, some metal compounds discriminate between bacterial targets and eukaryotic cells^64^. In this way, the material is toxic to *Candida albicans* cells, and at the same concentration does not cause damage FGH cells (Figs 4B, 5 and 6).

In this study, the viability and cytotoxicity of MB49 tumor and BALB/3T3 non-tumor cells exposed to the samples irradiated by different energy sources has been analyzed (Fig. 7). Our results show that the samples had effective abilities to cause the death of MB49 relative to normal BALB/3T3 cells, depending on the concentration (Fig. 7). The order of antitumor activity when the cells was exposed to a more effective concentrations (23.16 μg/mL) of the composites is: Ag NPs/α-Ag_2_WO_4_:NF > Ag NPs/α-Ag_2_WO_4_:E > Ag NPs/α-Ag_2_WO_4_:F > α-Ag_2_WO_4_. This is due to the formation of Ag NPs on the surface of the semiconductor and the generation of internal defects in the material, which cause a threshold between Ag toxicity and its antitumor activity. It was also observed that the material that was better for the antifungal activity, Ag NPs/α-Ag_2_WO_4_:F composite, showed no selectivity between tumor and non-tumor cells in relation to the others, because the number of Ag NPs on its surface exceeded its toxicity threshold^58^.

The samples are considered triggering elements for the cell death pathway. Oxidative stress induced by the samples linked to the intrinsic (mitochondrial effects) or extrinsic apoptotic network (cytoplasmic effect) is currently the most accepted description^65^, and many *in vitro* studies have identified a significant increase in ROS as a toxicity factor^66–68^, regulating the expression of apoptotic proteins (Figs 8 and 9). The signals that are produced in response to these stimuli increase the permeabilization of the mitochondria, interrupting the synthesis of ATP and releasing the pro-apoptotic molecules, which triggers the production of the protein complex, culminating with the final events of the cell death itself  ^65,69^.

In the case of Ag NPs/α-Ag_2_WO_4_ composites, the excited electrons from Ag NPs can be transferred to the conduction band (CB) of α-Ag_2_WO_4_ semiconductor and then further transferred to the noble metal particles owing to the Fermi-level equilibration. Under visible-light irradiation, the processes of the photo-induced charge separation and transfer at the interface of the metal Ag NPs and α-Ag_2_WO_4_ semiconductor can be divided into the following steps: (i) the visible light is harvested by the metal Ag, and then a hot electron transfer from Ag to α-Ag_2_WO_4_ takes place; (ii) the local electric field in the Ag NPs/α-Ag_2_WO_4_ composite can promote electron–hole pair separation under visible-light irradiation. The other Ag particles on the α-Ag_2_WO_4_ surface act as an efficient photocatalyst and trap the photoexcited electrons that come from the CB of α-Ag_2_WO_4._ The promotion effects on the separation of the photogenerated electron−hole pairs on α-Ag_2_WO_4_ by Ag can be achieved. Overall, the synergistic effect, enhanced visible-light absorption, and efficient electron–hole separation play an important role in enhancing the activity of Ag NPs/α-Ag_2_WO_4_ composite.

In the photocatalytic activity of both α -Ag_2_WO_4_ and Ag/α -Ag_2_WO_4_, Liu *et al*. proposed a mechanism involving three steps: (i) Light absorption and photoelectron excitation, i.e., the electrons are excited from the CB to the valence band (VB) and the photogenerated holes appear in the VB. (ii) Formation of the free radical. The photoelectrons at the CB at the surface of the semiconductor would react with the surrounding substances, such as O_2_ molecule and generate ${O^{\prime} }_{2}$ radical, which could turn into other ROS including $H{O}_{2}^{\ast }$, or H_2_O_2_ by successive photochemical processes. On the contrary, the photogenerated holes in the VB can yield OH^*^ by combining with *OH*^−^ (iii) The last step corresponds to radical oxidizing rearrangements involving ROS; it must be pointed out that neither α-Ag_2_WO_4_ nor Ag/α-Ag_2_WO_4_ could produce electron spin resonance (ESR) signals without light irradiation. However, after visible-light irradiation, the presence of radical OH^*^ is detected by ESR, where the ESR signals of the Ag/α-Ag_2_WO_4_ nanocomposites was almost 1.5 bigger than that of α-Ag_2_WO_4_, exhibiting a better photosensitive ability to produce OH^*^ under visible-light irradiation.

Avalos *et al*.^70^ measured the production of ROS induced by distinct sizes of Ag NPs in HepG2 and HL-60 tumor cells. The results showed that the higher ROS production can be attributed to an increment in the surface area of the NPs. In the work of Hussain *et al*.^71^ it was also demonstrated that for TiO_2_ NPs, the ROS modulation was proportional to the reactivity of the small area of the NPs. Depending of the magnitude of ROS production, the samples presented a different level of toxicity.

The Ag NPs/α-Ag_2_WO_4_:F composite has a major capacity to produce ROS (Fig. 9), which causes this material to have the best antifungal activity, as confirmed by assay (Fig. 4A). However, with respect to the antitumor activity, the high degree of cytotoxicity affects the tumor cell MB49 and non-tumor cell BALB/3T3 in the same way, indistinctly producing cell death at concentration of 23.16 µg/mL. On the contrary, the number of Ag NPs formed on the surface of Ag NPs/α-Ag_2_WO_4_:NF and Ag NPs/α-Ag_2_WO_4_:E composites is capable of producing a selective cytotoxic effect on tumor cells. This characteristic is probably related to the resistance to ROS action.

Thus, these electrons and holes ultimately produce ROS. As a function of the aerobic metabolism and oxidation of substrates, normal cells continuously produce ROS and the low level of intracellular ROS generated during the physiological activities acts as a factor in the differentiation, progression, arrest of growth, apoptosis, and immune response. However, when ROS are accumulated in a high quantity, the cells get into an oxidative stress state that induces a plethora of dysfunctional alterations in macromolecules such as DNA and lipids, among others that can lead to the cellular death. In order to counterbalance the excess of ROS production, the cells possess an efficient system formed by antioxidant enzymes such as superoxide dismutase (SOD), glutathione peroxidase, and catalase that protect the cell from oxidative stress^72^.

In the normal process, the redox state may contribute to tumor progression, increasing the expression of genes related to ROS metabolism, such as the enzyme SOD^73,74^. However, with exposure to materials, the induction of genotoxicity through DNA breakdown may occur. In this case, the production of ROS is an important factor. Materials have the potential to lead to cell death by the induction of autophagic dysfunction produced by mechanisms that lead to the overload or inhibition of lysosomal activity, interference of organelles trafficking by the cytoskeleton, and the breakdown of lysosomal stability (oxidative stress, alkalinization, osmotic edema, and membrane rupture). Finally, the materials induce the formation of the protein complex known as inflammassome that mediates the production of the IL-1β pyrogenic mediator, which stimulates the development of acute inflammatory process.

Here, we can assume that non-tumor cells could present some mechanism beyond tumor cells that allow survival when exposed to oxidative stress. This selectivity is associated with the basic mechanism that undergoes interference by the samples with the cells’ ability to compensate for the changes caused^75^. This is in contrast to the tumoral cells, in which the ROS act as secondary messengers in intracellular cascades, inducing and maintaining malignant phenotype. The DNA damage, mutations, and altered gene expression are involved in carcinogenesis^76^.

A large body of research about the microbial^53,54^ and antifungal^55^ activity of α-Ag_2_WO_4_ material has been presented by our research group, and very recently, we demonstrated that when the material is irradiated by pulsed fs laser, its bactericidal properties are enhanced^36^. The present study demonstrates, for first time, the antifungal and antitumor activity of Ag NPs/α-Ag_2_WO_4_ composite formed by electron beam and fs irradiation. However, it is important to remark that the mode through which these composites exert their biological actions has not as yet been fully elucidated. There is compelling evidence that ROS species are responsible for the antifungal and antitumor effects.

It is important to note that the elucidation of the mechanisms of action as well as the molecular changes caused in the cells is out of the aims of present work. Excellent reports on antifungal and potential tumoricidal NPs, particularly for realistic physiological situations, have been published in the literature. The ongoing works are based on different studies in which the production of ROS is the main mechanism of action of Ag containing materials for the elimination of neoplastic cells^63,77–84^. Tough, the researchers have widely explored the antibacterial efficacy of Ag NPs but their mode of action is still not clear^85,86^. Possible mechanisms contributing to the biological effects include both the direct damage to cell membranes by Ag NPs and the Ag NPs-mediated generation of ROS. He *et al*. point out that Ag NPs enhance a powerful oxidant through a reaction with H_2_O_2_, and the oxidizing species did not include the free OH^*^. It is well known that free OH^*^ is highly reactive and can damage virtually all types of macromolecules, especially the nucleic acid and lipids. This evidence indicates that the lack of significant generation of OH^*^ but large amount of other ROS species by the laser generated Ag NPs in our experiment suggest that the molecular mechanisms underlying the antifungal and antitumor effect might be dependent on the method used for the NP production and the target microbial species. It would be interesting in a future study to compare the behavior between the specific ROS species induced by electron beam and fs laser irradiation. This would further clarify the specific properties of the generated Ag NPs/α Ag_2_WO_4_ composite.

## Conclusions

The development and use of hybrid nanomaterials, composed by noble metal nanoparticles–semiconductor composites, are playing increasingly important roles and becoming useful multifunctional materials toward the development of advanced intelligent devices with novel, multiple, and versatile bioactivities.

In the current work, we deployed our investigations in this regard by constructing an interesting composite system formed by Ag NPs and α-Ag_2_WO_4_ fabricated by electron beam and fs laser irradiation on α-Ag_2_WO_4_, and its remarkable higher performance in antifungal and antitumor activity were demonstrated.

The synergistic effect of the plasmonic Ag nanoparticles on the α- Ag_2_WO_4_ semiconductor enhances the antifungal and antitumor efficiency. For the antitumor activity, the best results were achieved by the α-Ag_2_WO_4_:NF and α-Ag_2_WO_4_:E materials, which showed greater selectivity and efficacy against the action of MB49 tumor cells and greater cell viability of healthy BALB/3T3 cells. In the case of the antifungal activity, the synergism between the bacteriostatic effect of W and the higher oxidizing power of Ag NPs makes the α-Ag_2_WO_4_:F the best fungicide agent, with a minimum concentration of 3.90 µg/mL.

The irradiation of solids with energetic species, such as electrons or photons, normally gives rise to formation of atomic defects in the target and spoils the material properties. However, in spite of the damage, irradiation may overall have a beneficial effect on the target. This application motivated further studies of defect production under irradiation, because each implanted atom creates many lattice defects in the sample, imparting to the material new properties to be explored.

## Experimental Section

### Synthesis

The synthesis method, based on the aqueous coprecipitation method, of α- Ag_2_WO_4_ was previously reported by Longo *et al*.^36^.

### Electron irradiation

To irradiate the material with electrons to obtain Ag NPs/α-Ag_2_WO_4_:E composite, the α-Ag_2_WO_4_ sample was placed in a field emission gun scanning electron microscope (SEM-FEG) using a Supra 35-VP (Carl Zeiss, Germany) with an acceleration voltage of 15 kV for 5 min.

### Fs irradiation

α-Ag_2_WO_4_ pellets were irradiated with a Ti:sapphire laser (Femtopower Compact Pro, Femto Lasers) using 30 fs full width at half maximum (FWHM) pulses at the central wavelength of 800 nm, and a repetition rate of 1 kHz. To get more precise pulse compression at the sample, a programmable acousto-optic filter (DAZZLER, Faslite) was used. A laser beam of 6 mm diameter and mean power of 200 mW was focused onto the surface of a pellet target of α- Ag_2_WO_4_ with a 75 mm lens. To obtain the Ag NPs/α-Ag_2_WO_4_:F composite, the α- Ag_2_WO_4_ pellet was placed at the bottom of a quartz cuvette attached to a two-dimensional motion-controlled stage moving in a raster scanning at a constant speed of 0.45 mm/s in the focus plane perpendicular to the laser beam. At the focal position, the spot size was approximately 21 µm with a fluence of approximately 60 J/cm^2^. For the Ag NPs/α-Ag_2_WO_4_:NF composite, the position of the pellet was moved 8 mm closer to the convex lens to obtain a focal spot of approximately 84 µm and a fluence of approximately 3.6 J/cm^2^. The large difference in the fluence over the two samples is the key parameter for the structural differences between them as, reported in^38^.

### Characterization

The microcrystalline powders were characterized using XRD with a D/Max-2500PC diffractometer (Rigaku, Japan) involving CuK α radiation (λ = 1.54056 Å) in the 10–70° 2θ range at a scan rate of 0.01° min^−1^. Micro-Raman spectroscopy was carried out using an iHR550 spectrometer (Horiba Jobin-Yvon, Japan) coupled to a charge-coupled device (CCD) detector and an argon-ion laser (MellesGriot, USA) operating at 633 nm with a maximum power of 200 mW. The spectra were measured in the 50–1000 cm^−1^ range. The SEM images were analyzed using field emission gun scanning electron microscopy (FEG-SEM) on an FEI instrument (Model Inspect F50) operating at 5 kV.

### Antifungal study

The culture of *Candida albicans* ATCC 90028 was kept frozen at −80 °C. Before the tests, the cells were thawed and streaked onto Sabouraud dextrose agar supplemented with 0.05 g/L chloramphenicol (SDA, Acumedia Manufacturers Inc., Baltimore, MD, USA). The plate was incubated for 24 h at 37 °C, and then five colonies were transferred to yeast nitrogen base culture medium supplemented with 100 mM glucose (YNB) and incubated for 16 h to prepare the pre-inoculum. Sequentially, the pre-inoculum was diluted 1:10 in fresh YNB and incubated for an additional 8 h until the cells had the mid-log phase of growth. The cells were standardized at a concentration of 10^7^ by a spectrophotometer and incubated for 90 min for the initial cell adhesion phase at 37 °C. Then, the non-adhered cells were removed through two washes with phosphate-buffered saline solution (PBS, pH 7.2), and incubated for 24 h with the samples two-fold diluted in YNB for the biofilm formation phase.

After the growth of the biofilm in contact with the particles, 10-fold dilutions were prepared of each well and 10 µL aliquots of these were inoculated in duplicate SDA. The plates were incubated for 24 h at 37 °C, the colony forming units per milliliter (CFU/mL) were calculated and log_10_-transformed.

### Biocompatibility of antifungal Ag NPs/Ag_2_WO_4_:NF and Ag NPs/Ag_2_WO_4_:F composites

For biocompatibility assays, human gingival fibroblasts (FGH cell line from Rio de Janeiro Cell Bank Code 0089) were used as a model in monolayer cultures. The cells were cultured in Dulbecco’s Modified Eagle’s Medium Low Glucose (DMEM, Sigma Chemical Co., St. Louis, MO, USA) medium, supplemented with 10% fetal bovine serum (FBS, Gibco, Grand Island, NY, USA), 100 IU/mL penicillin, 100 mg/mL streptomycin (Sigma-Aldrich, St. Louis, MO, USA), and 2 mM L-glutamine (Gibco, Grand Island, NY, USA) in a humidified atmosphere containing 5% CO_2_ at 37 °C. After reaching 90% confluence, the cells were washed with PBS, recovered using trypsin, and re suspended in fresh medium prior to further analyses. The assessment of α- Ag NPs/Ag_2_WO_4_:NF and Ag NPs/α-Ag_2_WO_4_:F composites on cell viability were evaluated by the resazurin assay (Alamar Blue quantitative fluorimetric assay). FGH cells were seeded at 8 × 10^3^ cells/well directly into the 96-well polystyrene black plate (TPP Tissue Culture Plates, USA) for monolayer cell culture. The cells were incubated at 37 °C under 5% CO_2_ for 24 h. Next, the medium was removed and 200 μL of the fresh medium containing the composites was added at 3.90, 7,81, 15.62, and 31.25 µg/mL. In sequence, 10% of resazurin assay-Alamar Blue (Invitrogen, Carlsbad, CA, USA) was added into each well and the fluorescence signals were measured using a Fluoroskan (Fluoroskan Ascent FL; Thermo Scientific; Waltham, MA, USA) at an excitation wavelength at 544 nm and an emission wavelength at 590 nm, after 24-h incubation. The experiment was performed in triplicate and with three independent biological repetitions. To confirm the impact of the concentrations of the samples on gingival fibroblasts behavior we investigated the proliferation/cell death staining by labeling the cells with CellTracerTMCFSE (Invitrogen, Eugene, OR,USA) and propidium iodide - PI 20 µM (Invitrogen, Eugene, OR,USA) after 24 h of incubation with materials. This assay was performed by CLSM using a Carl Zeiss LSM 800 microscope (Zeiss, Jena, Germany). The green fluorescence of CFSE and the red one of PI were employed by laser excitation at 488 nm and 561 nm, respectively. Images were acquired through 20X dry (Plan NeoFluar NA). Finally, the cell morphology was investigated by SEM using a JEOL JSM-6610LV microscope (Ref JEOL). First, 5 × 10^4^ cells were plated on sterile cover glass discs on a 24-well plate (TPP Tissue Culture, Switzerland) and maintained at 37 °C under 5% CO_2_ conditions. After 24 h the medium was removed and materials were added at concentrations 3.90, 7,81, 15.62, and 31.25 µg/mL for 24 h. After this period, the samples were prepared for SEM analyses. A fixation step was performed by sample incubation in a solution of 2.5% glutaraldehyde (pH 7.4) at room temperature for 1 h. The PBS-washed discs were then subjected to a standard procedure for dehydrating of specimens: 70% and 90% ethanol for 1 h per step, ending with five changes within 30 minutes of 100% ethanol. Prior to visualization, the discs were placed under *vacuum* to protect the dry samples from moisture, and after storage for seven days, the discs were sputter-coated with gold. The experiment was performed in duplicate for each experimental and control group, which were: CT: cells in standard culture conditions, cell death controls for Alamar Blue and SEM: Triton-X 100 0.9% buffer (LB) or 10 µM of Camptothecin for 8 h (CAM) for CLSM.

### Cell viability MB49 and BALB/3T3 on α-Ag_2_WO_4_ samples exposure

To investigate the *in vitro* toxicity of the samples, the test was carried out with the cell lines MB49 (tumor cell) and BALB/3T3 (non-tumor cell). Both cell lines were plated on 2 96-well culture plates (Corning Incorporated, NY, USA) to a concentration of 1.10^5^ cell/well in DMEM medium (SIGMA-ALDRICH, USA) in the presence of L-glutamine (2 mmol L^−1^) and penicillin/streptomycin (100 U mL^−1^) with the addition of fetal bovine serum (10% SFB) and kept overnight. The cells were exposed to α-Ag_2_WO_4_ samples irradiated by different sources at the following concentrations: 4.63, 11.58, 23.16 and 46.31 µM and a negative control. After the 24 h exposure, the supernatant containing the nanomaterials was collected and the resulting cells were washed with PBS (1X) buffer and the cell viability test was performed by the resazurin assay-Alamar Blue (SIGMA-ALDRICH, USA), was added into each well and the fluorescence signals were measured using a Fluoroskan (Fluoroskan Ascent FL; Thermo Scientific; Waltham, MA, USA), according to the instructions provided by the supplier. The readings were taken using a spectrophotometer with absorbance in the range 570–600 nm. The experiment was performed in triplicate.

### Reactive oxygen species generation assay

The MB49 and BALB/3T3 cells were plated under the same conditions described in item 1, and exposed to the materials at the concentration that presented the best results in the cell viability assay. For the measurement of the production of ROS, kinetics (ROS production/time) were performed for 105 min (every 15 min) using 100 μm 2′,7′-dichlorofluorescein diacetate (DCF-DA, SIGMA-ALDRICH, USA). The measurement of the ROS production was performed using a Spectra Max i3 (Molecular Devices) with 485–530 nm excitation.

### Cytotoxicity assay: apoptosis and necrosis

The MB49 and BALB/3T3 cells were plated in black plate 96-well (Corning Incorporated, NY, USA) under the same conditions described above, and exposed to the materials at the concentration that showed the best results in the previous assay. After the 24 h exposure, the cells were washed with PBS buffer (1X) and subjected to the apoptosis and necrosis assay using acridine orange/ethidium bromide (AO/EB), according to the instructions provided by the supplier. The total count of apoptotic and necrotic cells, as well as the analysis of cellular morphology, was performed on the ImageXpress Micro (Molecular Device) with 515–560 nm excitation filter and 590 nm barrier filter.

### Scavengers tests

The scavengers test was performed from the degradation of rhodamine B (RhB) (Aldrich 95%) under ultraviolet light. We dispersed the samples (50 mg) in a RhB solution (50 mL 1 × 10^−5^ mol L^−1^) in a beaker placed in an ultrasonic bath (Branson, model 1510; frequency 42 kHz) for 10 min. This solution was maintained in the dark, under stirring for 30 min to allow the adsorption–desorption process. After this process, the initial aliquot was withdrawn. Next, these solutions were illuminated by four visible lamps (Philips TL-D, 15 W) in a photocatalytic system maintained at 20 °C in a thermostatic bath under stirring. The final aliquot was withdrawn after 60 min. This process was repeated for all the samples. The aliquots were centrifuged to obtain the liquid phase alone. Variations in the absorption band maximum at λ = 553 nm (RhB) were measured by performing UV-Vis absorption spectroscopy measurements of the solution on a V-660 spectrophotometer (JASCO). To analyze the action of the radicals OH^*^ and ${O^{\prime} }_{2}$, we performed tests by adding appropriate reactive species scavengers such as 0.067 g of BQ (Alfa Aesar) and 0.0589 mL tert-butyl alcohol (TBA), respectively.

### Data presentation and statistical analyses

Data were represented by the mean and standard error of the mean. The data followed the normal distribution; according to the Shapiro-Wilk test, differences between groups were determined using the ANOVA and Newman–Keuls tests for multiple comparisons. The nominal variables were analyzed by Fisher’s exact test. The software used for analyses was GraphPad Prism version 5.00 for Windows (GraphPad Software, San Diego,CA, USA). The tests were considered statistically significant when the p-value was less than 0.05.
